# Rapid measurement of radical oxygen species in biological fluids may help to diagnose infection

**DOI:** 10.1186/2197-425X-3-S1-A645

**Published:** 2015-10-01

**Authors:** AC Lukaszewicz, J Bardon, V Faivre, B Huot, D Payen

**Affiliations:** Département d'Anesthésie Réanimation SMUR, Hopital Lariboisiere, Assistance Publique Hôpitaux de Paris, Paris, France; Sorbonne Paris Cité, Université Paris Diderot, Paris, France; INSERM UMR 1160, Paris, France

## Introduction

Recently, we showed the interest of instantaneous production of radical oxygen species (ROS) for diagnosis of meningitis ([1]). Polymorphonuclear neutrophils (PMN) are a major source of ROS through the activation of protein kinase C and NADPH oxidase pathway, which may differ according to stimuli.

## Objectives

To test if ROS measurements related to infection is different from other cause of ROS production in: pleural (PlF), peritoneal (PerF) fluids and bronchoalveolar lavage (BAL).

## Methods

Monocentric study in ICU patients with systemic inflammatory response syndrome and suspicion of infection. PlF, PerF or BAL sampled for measurement of ROS by luminescence (luminol, basal condition or stimulation by phorbol 12-myriaste 13-acetate (PMA, PKC activator)) ([1]). Non parametric tests, p < 0.05. Results are expressed in area under the curve of luminescence (AUC), median (25-75th percentiles).

Definition of infection: microbiological positivity in BAL culture (≥10^4^ UFC/ml), in PerF or PlF, or number of PMNs ≥250/mm^3^ in PerL

## Results

58 patients, SAPSII 43 (34-51), temperature 37.5°C (36.6-38), leukocytes 13800/mm^3^ (11175-23800). 17 PerF (35% infected), 28 PlF (29%infected), 20 BAL (35% infected). Culture were positive for *Stenotrophomonas maltophilia* in PerF and *Pseudomonas aeruginosa* in PlF and BAL.

AUC ROS luminescence was elevated for basal (Figure) and PMA stimulated condition when infection was present (grey boxes) in PerF (p = 0.0009, p = 0.001 respectively) and in PlF (p = 0.0016, p = 0.0019) compared to negative culture (white boxes), but not in BAL. If ROS production was reported to the number of PMNs, there was no difference according to infection.

**Figure 1:**
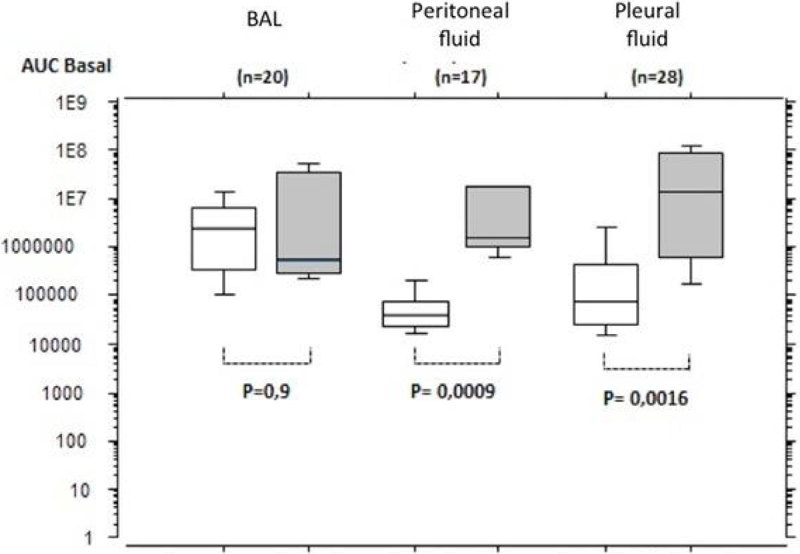
Figure 1

## Conclusions

Instantaneous production of ROS was significantly increased when infection was present in PerF and PlF but not in BAL. These results questioned the validity of BAL for characterization of inflammation ([2]). This test is simple to diagnose the presence of infection and requires a validation in a larger cohort.

## GRANT ACKNOWLEDGMENT

Ministère de l´Enseignement Supérieur et de la Recherche

Patent WO 2012/014156
